# Novel Thienyl-Based Tyrosine Kinase Inhibitors for the Treatment of Hepatocellular Carcinoma

**DOI:** 10.3390/jpm12050738

**Published:** 2022-05-01

**Authors:** Andi Ma, Bernhard Biersack, Nils Goehringer, Bianca Nitzsche, Michael Höpfner

**Affiliations:** 1 Institute of Physiology, Charité—Universitätsmedizin Berlin, Corporate Member of Freie Universität Berlin, Humboldt-Universität zu Berlin and Berlin Institute of Health, 10117 Berlin, Germany; andi.ma@charite.de (A.M.); nils.goehringer@charite.de (N.G.); michael.hoepfner@charite.de (M.H.); 2 Organic Chemistry 1, University of Bayreuth, Universitätsstrasse 30, 95440 Bayreuth, Germany; bernhard.biersack@uni-bayreuth.de

**Keywords:** hepatocellular carcinoma, anticancer drugs, treatment, angiogenesis, multi-kinase inhibitor

## Abstract

New medical treatments are urgently needed for advanced hepatocellular carcinoma (HCC). Recently, we showed the anticancer effects of novel thiophene-based kinase inhibitors. In this study, we further characterized the antineoplastic effects and modes of action of the two most promising inhibitors, Thio-Iva and Thio-Dam, and compared their effects with the clinically relevant multi-kinase inhibitor, sorafenib, in HCC cells. Crystal violet staining and real-time cell growth monitoring showed pronounced antiproliferative effects in Huh-7 and SNU-449 cells with IC_50_ values in the (sub-)micromolar range. Long-term incubation experiments revealed the reduced clonogenicity of Thio-Iva and Thio-Dam-treated HCC cells. LDH-release tests excluded cytotoxicity as an unspecific mode of action of the inhibitors, while flow cytometry analysis revealed a dose-dependent and pronounced G2/M phase cell cycle arrest and cyclin B1 suppression. Additionally, mitochondria-driven apoptosis was observed through the cytosolic increase of reactive oxygen species, a concomitant PARP cleavage, and caspase-3 induction. Both compounds were found to effectively inhibit the capillary tube formation of endothelial EA.hy926 cells in vitro, pointing towards additional antiangiogenic effects. Antiangiogenic and antineoplastic effects were confirmed in vivo by CAM assays. In summary, the thienyl-acrylonitrile derivatives, Thio-Iva and Thio-Dam, exert significant antineoplastic and antiangiogenic effects in HCC cells.

## 1. Introduction

Hepatocellular carcinoma (HCC) is the sixth most common cancer in the world [1]. HCC emerges in patients with chronic liver inflammation associated with viral infection, alcohol abuse, or metabolic syndrome. Its incidence is constantly rising, and the relative 5-year survival rate is below 20%. At present, the clinical treatment options for early-stage HCC include surgical resection, liver transplantation, or percutaneous ablation [3]. However, as most patients are already in an advanced disease stage when diagnosed, these radical treatment options are often not applicable. For patients with advanced HCC, palliative therapy by trans-arterial chemoembolization or systemic therapy with tyrosine kinase inhibitors such as sorafenib are methods of choice. However, tumor growth control, relieve of disease-related symptoms, and the overall survival of sorafenib-treated patients with advanced HCC is not convincingly improved and is hampered by the occurrence of resistance to sorafenib treatment [4]. Thus far, no effective medical treatment exists for patients suffering from advanced HCC, emphasizing the urgent need for new and efficient therapeutic agents for HCC treatment.

Key enzymes of cellular signal transduction pathways correlated with tumor cell differentiation and proliferation are valuable targets for anticancer drug screening leading to the development of new drug candidates with high efficiency, low toxicity, and high specificity [5].

Sorafenib is a prominent clinically approved example of a small molecule multi-kinase inhibitor, which targets vascular endothelial growth factor receptors (VEGFR) 1–3, platelet-derived growth factor receptor-β (PDGFR-β), and rapidly accelerated fibrosarcoma kinases (Raf kinases) [6]. Sorafenib was the only first-line systemic targeted drug available for advanced HCC for almost one decade, with a survival benefit of three months [7]. 

However, clinical studies have reported that a considerable proportion of HCC patients does not respond to sorafenib treatment. The response rate to sorafenib is less than 50%, and most patients develop disease progression within six months [8,9]. Due to the early occurrence of sorafenib resistance, most patients do not have long-term benefits, and thus the overall efficacy of sorafenib is far from satisfactory. Over the last years, further first-line and second-line therapies which are based on the receptor tyrosine kinase inhibitors regorafenib, lenvatinib, or ramucirumab have emerged [10,11,12]. Immunotherapy is a relatively new field of HCC research. The majority of HCCs arise from chronic liver disease where T cells are constantly exposed to antigen and inflammatory signals. This condition induces a state of upregulated receptors, such as the programmed cell death protein-1 (PD-1). The PD-1 inhibitor nivolumab was approved in 2017 as a second-line treatment for advanced HCC but failed to show statistically significant benefits over sorafenib such as being progression-free and improved overall survival rates [13]. These drawbacks of HCC immunotherapies necessitate stronger efforts in the search for new drugs against HCC. 

Tumor angiogenesis plays a vital role in the growth and dissemination of solid tumors. Hypervascularity and marked vascular abnormalities such as arterialization and sinusoidal capillarization are common tumor-associated features of HCC [14]. Vascular endothelial growth factors (VEGFs, e.g., VEGF-A, VEGF-B, VEGF-C, VEGF-D) and the receptor tyrosine kinases (RTKs) VEGFR-1, VEGFR-2, and VEGFR-3 are crucial for the promotion of tumor angiogenesis [15,16]. Tumor-induced angiogenesis is based on two mechanisms, i.e., the overexpression of angiogenic factors and the inhibition of anti-angiogenic factors, which lead to the enhanced development of blood vessels lacking in normal vascular structures with regulated blood vessel diameter and tissue-related perfusion. Antiangiogenic therapy based on antibodies and small-molecule VEGFR inhibitors was developed to inhibit the growth and further spreading of abnormal tumor blood vessels that lead to tumor hypoxia and shrinkage [17,18].

**Figure 1:**
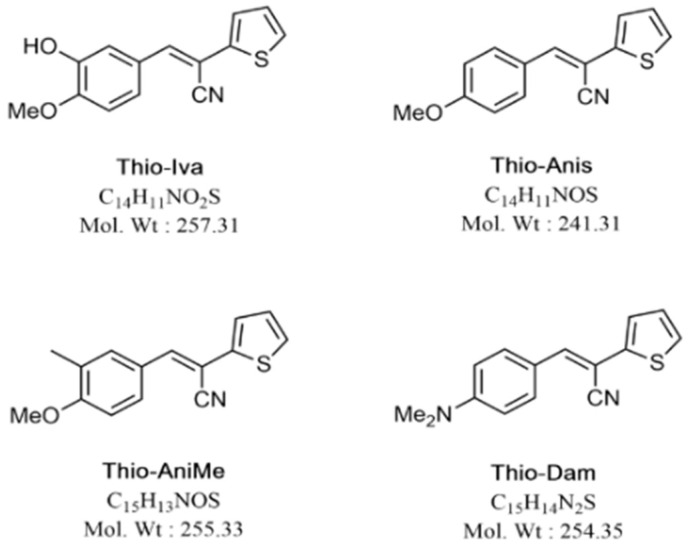
Chemical structures of E-2-(2-thienyl)-3-acrylonitrile RTK inhibitors used in this study.

Various reports have described the promising anti-tumor activities of thiophene-based compounds [19,20]. Recently, we identified some *E*-2-(2-thienyl)-3-acrylonitrile derivatives with high anti-tumor efficacy in p53 wild-type HepG2 hepatoblastoma cells which were more active than the clinically applied multi-kinase inhibitor sorafenib [21]. These compounds are likewise multi-kinase inhibitors with preferential activity against VEGFR-2. In this work, we further analyzed the mode of action of these promising compounds (Figure 1) in HCC cells.

## 2. Materials and Methods

### 2.1. Compounds

Stock solutions (10 mM) of Thio-Iva, Thio-Dam, Thio-Anis, Thio-Anime, and sorafenib were prepared in dimethyl sulfoxide (DMSO Thermo Fisher Scientific, Inc., Waltham, Ma, USA) and stored at −20 °C. Sorafenib was purchased from Targetmol (T0093L, Boston, MA, USA). Thio-Iva, Thio-Dam, Thio-Anis, and Thio-Anime were synthesized and provided by Dr. Biersack (Dept. of Organic Chemistry, University of Bayreuth, Bayreuth, Germany) [21].

### 2.2. Biological Evaluation

#### 2.2.1. Cell Culture 

Highly differentiated and p53-mutated Huh-7 (JCRB#0403) human hepatocellular carcinoma cells were grown in RPMI 1640 medium supplemented with 10% fetal bovine serum, 100 U/mL penicillin, and 100 mg/mL streptomycin (all from Gibco, Thermo Fisher Scientific, Inc., Waltham, Ma, USA). The p53-mutated SNU-449 cells (ATCC#2234) were grown in RPMI 1640 medium supplemented with 10% fetal bovine serum, 100 U/mL penicillin, 100 mg/mL streptomycin, 1%HEPES, and 1% sodium pyruvate. EA.hy926 human umbilical vein cells were grown in DMEM containing 10% fetal bovine serum, 100 U/mL penicillin, and 100 mg/mL streptomycin. All cells were incubated at 37 °C, 5% CO_2_, 95% humidified atmosphere.

#### 2.2.2. Crystal Violet Staining

The treatment-induced inhibition of cell proliferation was assessed using crystal violet staining, as described earlier [22]. In brief, 1500 cells/well seeded in 96-well plates were allowed to adhere to the bottom of the wells for 72 h. Thereafter, the cells were incubated with rising concentrations (0.1–20 μM) of each test compound for up to 48 h. After that, cells were rinsed with PBS, fixed with 1% glutaraldehyde, and 0.1% crystal violet (N-hexamethylpararosaniline, Sigma Aldrich) was added to stain the cells. Unbound dye was removed by rinsing with water. The cell-bound crystal violet was dissolved using 0.2% Triton X-100 (Sigma-Aldrich, Munich, Germany). The extinction of crystal violet, which increases linearly with the increase of the cell number, was measured with an ELISA-Reader (Dynex Technologies, Denkendorf, Germany) at 570 nm [23]. 

#### 2.2.3. Real-Time Monitoring of Cell Proliferation

The real-time cell analyzer iCELLigence system (ACEA Biosciences San Diego, CA, USA) was used to monitor cell proliferation and survival, as previously described [24]. Cells were seeded in 8-well micro-E-plates (ACEA Biosciences, San Diego, CA, USA) at a density of 6000 cells/well. Incubation for 24 h allowed for attachment, and the medium was replaced thereafter by Thio-Iva or Thio-Dam-containing medium in rising concentrations (0.1–10 µM). The impedance-based iCELLigence system determined proliferation by measuring changes in the electrical resistance of the bottom of each well every 15 min, for up to 96 h. Electrical resistance increases when the number of attached cells increases due to mitosis. Data are recorded as a unitless parameter called cell index, which is defined as (R_tn_–R_t0_)/4.6 Ohm, with R_tn_ being the measured resistance at time point n and R_t0_ being background resistance measured at time point T0.

#### 2.2.4. Colony Formation Assay

The proliferation of long-term effects was assessed by colony formation assays. Cells were seeded in 6-well plates at a density of 300 cells/well, and colony formation and growth were observed for 2 weeks [21]. Then, the colonies were washed twice with PBS and fixed with 4% formaldehyde for 1 h before staining with 0.5% crystal violet for 3 min. A colony was defined as a cell aggregate with 50 or more cells [21], and so only colonies with 50 or more cells were counted. Representative images were taken by a kappa digital camera system (Kappa Optronics GmbH, Gleichen, Germany). Stained colonies were quantified using the Colony Area ImageJ plug-in application (Vision 1.52a, National Institutes of Health, USA). 

#### 2.2.5. Enzymatic Kinase Assay

A cell-free kinase assay consisting of a custom panel of 32 protein kinases involved in cell proliferation and angiogenesis was used to screen the kinase-inhibiting potency of Thio-Iva (10 µM). The assay was performed by Eurofins Kinase Profiler TM service (Eurofins, Celle-Lévescault, France), and the determination of enzymatic activity was assessed as previously described [21]. Moreover, a dose-response curve for Thio-Iva (0.003–30 µM) was executed to determine the IC_50_ of Thio-Iva-induced VEGFR-2 inhibition.

#### 2.2.6. Determination of Caspase-3 

Caspase-3 activity was measured to determine Thio-Iva and Thio-Dam-induced apoptosis in HCC cells [25]. A total of 100,000 cells/well were seeded in 6-well plates and maintained for 24 h. Thereafter, the cells were incubated with 1 μM and 10 μM of each test compound for 24 h and 48 h, respectively. After that, the cells were collected and lysed using lysis buffer at 4 °C for 30 min. The protein content of the samples was quantified using Pierce^TM^ BCA Protein Assay Kit (Thermo Fisher Scientific) to adjust equal amounts of protein for the following caspase-3 determination. The samples were incubated with AC-DEVD-AMC (EMD Millipore, Billerica, MA, USA) at 37 °C for 1 h. Active caspase-3 cleaved AC-DEVD-AMC to produce fluorescent AC-DEVD, which was measured using a VarioSkan Flash 40053 microplate luminometer (Thermo Fisher Scientific, Waltham, MA, USA; filter sets: e.g., 360/40 nm, em 460/10 nm) [21].

#### 2.2.7. Determination of Compound-Induced Cytotoxicity 

Cytotoxicity was quantified with a KitPLUS lactate dehydrogenase (LDH) assay (Roche Diagnostics GmbH, Mannheim, Germany). A total of 4 × 10^4^ cells/well were seeded in 96-well plates for 24 h and then incubated with 1 μM and 10 μM of Thio-Iva and Thio-Dam for 6 h and 24 h. Supernatant was collected for LDH determination according to the manufacturer’s instructions. Then, 100 μL of a mixture of catalyst and dye solution was added, and cells were incubated for a maximum of 30 min. LDH can catalyze the synthesis of pyruvate from lactic acid, and then the reaction of pyruvate to 2,4-dinitrophenylhydrazine, which forms a brownish red solution under basic conditions. An ELISA reader (Dynex Technologies, Denkendorf, Germany) was used at 490/630 nm for the measurement of cytotoxicity indicating the leakage of LDH into the supernatant of the cells. Data are expressed as the percentage (%) of the total LDH activity (LDH in the medium + LDH in the cell), according to the equation % LDH released = (LDH activity in the medium/total LDH activity) × 100 [23].

#### 2.2.8. Scratch Assay 

Cells (1.5 × 10^5^ cells/well) were seeded in 6-well pates and allowed to grow to (sub-) confluence. Using a 10 µL pipette tip, the cell monolayer was scratched vertically. The cells on the edge of this artificial gap migrate into the cell-free area to close the gap in a time-dependent manner. The cells were rinsed with PBS, and fresh medium was added, which contained rising concentrations of Thio-Iva and Thio-Dam (1–10 µM), and a corresponding volume of DMSO was used for control. The cells were incubated for 24 h (37 °C, 5% CO_2_, 95% humidity), followed by photographical documentation with an EVOS M5000 microscope (Thermo Fischer Scientific, Waltham, MA, USA). The migration of cells was quantified using the TScratch software (https://github.com/cselab/TScratch, accessed on 5 April 2022; CSElab, Zurich, Swiss). Migration values were normalized to control, which was set as 100% [25].

#### 2.2.9. Measurement of Reactive Oxygen Species (ROS) 

The formation of cytosolic ROS was performed as described [26]. Measurement was performed by using the membrane-permeable dye CellROX^®^ Orange (Thermo Fisher Scientific, Inc., Waltham, Ma, USA), which accumulates in the cytoplasm and exhibits a strong fluorescent signal upon oxidation at excitation/emission levels of 545 nm/565 nm [26]. CellROX^®^ Orange reagent (1 μM) was applied together with Thio-Iva (1 and 10 µM) and Thio-Dam (1 and 10 µM). Cells incubated with 1.6 mM H_2_O_2_ for 30 min served as positive controls. Formation of ROS was measured after 24 h of incubation with the compounds using a ZOE^TM^ Fluorescent Cell Imager (Biorad, Munich, Germany). 

#### 2.2.10. Cell-Cycle Analysis by Flow Cytometry

Flow cytometry was applied for cell cycle analysis by staining the DNA of treated HCC cells with propidium iodide (PI) nucleic acid stain (Invitrogen, Eugene, OR, USA). Huh-7 and SNU-449 cells were seeded in 6-well plates with a density of 20,000 cells/mL and treated with different concentrations of Thio-Iva and Thio-Dam for 48 h. Then, the cells were harvested and fixed in 70% ethanol overnight and incubated with RNaseA (0.4 mg/mL) in PBS at 37 °C for 30 min. PI was added and cells were incubated for 30 min in the dark. Samples were analyzed using FACSCanto II (BD Biosciences, Heidelberg, Germany). Data analysis was done with FlowJo 10.4 software (LLC, Ashland, OR, USA). 

#### 2.2.11. Tube Formation

A total of 50 μL of cold Matrigel/well (Corning™ 354234, Tewksbury, MA, USA) was plated out in a 4 °C cold 96-well plate and allowed to polymerize in a 37 °C incubator for 2 h. Then, EA.hy926 cells were suspended in DMEM containing different concentrations of Thio-Iva and Thio-Dam and inoculated to the Matrigel at a density of 2500 cells/well. After incubation at 37 °C and 5% CO_2_ for 6 h, photographical documentation was executed with an EVOS M5000 microscope (Thermo Fischer Scientific, Waltham, MA, USA). For the analysis and quantification of tube formation, the Angiogenesis Analyzer plugin of ImageJ (NIH, Bethesda, MD, USA) was employed. Results are expressed as total segment length. 

#### 2.2.12. Western Blot 

Western blotting was performed as described previously [27]. In short, radioimmunoprecipitation assay (RIPA) buffer was used to lyse whole-cell extracts. Protein was quantified by the bicinchoninic acid (BCA) assay. An equal amount of protein (20 µL) was separated from each sample by sodium dodecyl sulfate-polyacrylamide gel electrophoresis and transferred to a polyvinylidene fluoride membrane (PVDF), followed by incubation with primary antibody overnight at 4 °C. Antibodies of phospho-VEGF Receptor2 (#3817 Cell Signaling Technology, Danvers, MA, USA, 1:500), cyclin B1 (sc-245 Santa Cruz Biotechnology, Santa Cruz, CA, USA, 1:1000), poly-(ADP-ribose)-polymerase (PARP) and cleaved PARP (11835238 Roche Mannheim, Germany, 1:1000), and β-actin (A5441 Sigma Aldrich, Taufkirchen, Germany, 1:2000) were used. Then, incubation with the corresponding anti-mouse (NA931VS Santa Cruz Biotechnology, 1:10,000) or anti-rabbit (NA934VS Santa Cruz Biotechnology, 1:10,000) peroxidase-coupled anti-IgG secondary antibodies was carried out at room temperature for a minimum of 1 h. Subsequently, antibody bondage was illustrated using Clarity Max ECL Western Blotting Substrates (Biorad, Munich, Germany) for detection and Celvin-S developer (Biostep, software SnapAndGo, Burkhardtsdorf, Germany) for development.

#### 2.2.13. Chicken Chorioallantoic Membrane Assay (CAM)

In vivo assays employing the chorioallantoic membrane (CAM) of fertilized chicken eggs were performed to test the anti-neoplastic and anti-angiogenic effects of the novel compounds, as described previously [21]. In short, fertilized chicken eggs (Gallus gallus) were obtained from a commercial provider (Valo Biomedia GmbH, Osterholz-Scharmbeck, Germany), and the development was induced by incubating the eggs at a temperature of 37.8 °C with 66% relative humidity. On day 3, the eggs were opened by cutting the shell at the top part of the egg.

For anti-angiogenesis testing, a silicone ring (diameter: 5 mm) was placed on the CAM for 24 h to be able to stably connect the ring to the CAM. On day 12, 20 µL of test compounds (0.2, 0.5, 1.0 µM) diluted with 0.9% NaCl was pipetted into the ring. The blood vessel status of the CAM was documented after 48 h by using a digital camera (Distelkamp-Electronic, Kaiserslautern, Germany). The degree of angiogenesis was quantified by measuring the length of the blood vessels using Image Pro Plus 6.0 (Image-pro Plus, Media Cybernetics, Inc., Silver Spring, MD, USA). 

For anti-neoplastic testing, a total of 3 × 10^6^ Huh-7 cells were resuspended in 10 µL cell culture media and 10 µL Matrigel (Corning™ 354234, MA, Tewksbury, USA) (BD Biosciences) before the cell suspension was applied to a silicone ring (5 mm in diameter) on the CAM of fertilized chicken eggs on day 8 of their embryonic development. The tumor-bearing chicken eggs were incubated for 24 h at 37.8 °C to stimulate tumor formation, followed by the topical application of 20 µL medium containing Thio-Iva, Thio-Dam, or sorafenib. After an incubation period of 72 h at 37.8 °C and 66% humidity, the tumors were excised and carefully weighed to determine their mass. 

#### 2.2.14. Statistical Analysis

GraphPad (version 8.00, San Diego, CA, USA) was used for statistical analysis. Unless otherwise specified, all experiments were independently repeated for 3–5 times, and the results are expressed as means ± SD or SEM, respectively. Statistical significance was calculated by performing a one-way analysis of variance (ANOVA).

## 3. Results

### 3.1. Biological Evaluation

#### 3.1.1. IC_50_ Determination of Novel Thiophene-Based Compounds in HCC Cells

The crystal violet staining method was used to determine the growth inhibitory effects of the four thiophene-based test compounds on the two human HCC cell lines, Huh-7 and SNU-449. Thio-Iva showed the highest activities, with IC_50_ values of 0.29 ± 0.18 μM (Huh-7) and 0.53 ± 0.32 μM (SNU-449) after 48 h of treatment. Thio-Dam also showed considerable antiproliferative activity, with IC_50_ values of 0.81 ± 0.26 μM and 1.64 ± 0.51 μM, respectively, and thus, both Thio-Iva and Thio-Dam were distinctly more active than the clinically approved VEGFR inhibitor sorafenib (IC_50_ = 2.50 ± 0.14 μM for Huh-7 and >8 μM for SNU-449) (Table 1). The closely related derivatives Thio-Anis and Thio-AniMe were also slightly more active against Huh-7 cells than sorafenib. However, in the SNU-449 cells, Thio-Anis, Thio-AniMe, and sorafenib were distinctly less active and did not show pronounced antiproliferative activity at doses of 8 µM and higher. Thus, for further evaluations, only the two best working compounds, Thio-Iva, and Thio-Dam, were chosen. 

#### 3.1.2. Kinase Inhibitory Effects of Thio-Iva and Thio-Dam 

**Figure 2:**
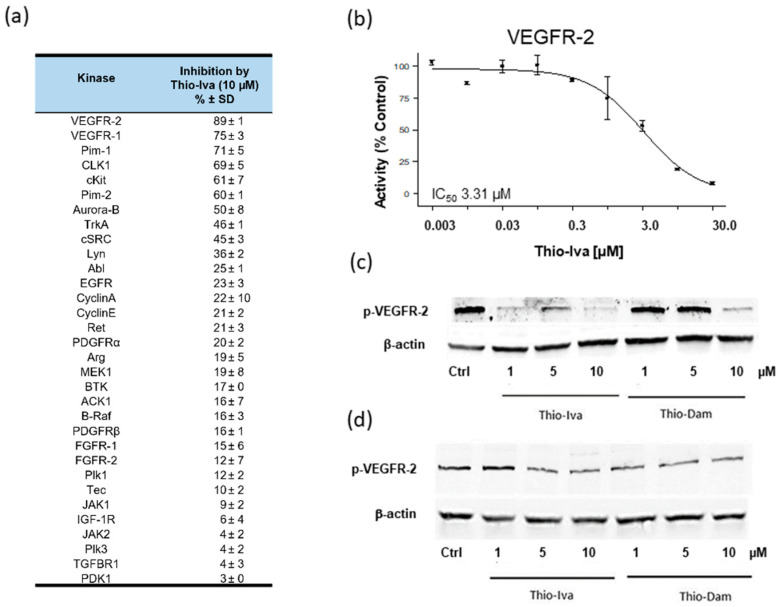
VEGFR-2 inhibition by novel compounds. Enzymatic kinase profiling on 32 kinases revealed multi-kinase inhibition of Thio-Iva, with the most pronounced effects on VEGFR-2 kinase (**a**). Dose–response curve for Thio-Iva-induced VEGFR-2 kinase inhibition (**b**). Data are given as means ± SD of *n* = 2–3 independent determinations per kinase. Western blot of Thio-Iva and Thio-Dam-induced inhibition of VEGFR-2 phosphorylation in Huh-7 (**c**) and SNU-449 (**d**) cells after 24 h of incubation, confirming the VEGFR-2 inhibiting effects of both compounds on the cellular level.

A cell-free kinase assay was used to screen for the kinase inhibitory potency of the most effective thiophene-based test compound, Thio-Iva, in a customized panel of 32 protein kinases. The kinases were selected because of their relevance to the proliferation and angiogenesis of HCC [21]. Thio-Iva showed multi-kinase inhibitory activity, with the most pronounced effects on VEGFR-2, which was inhibited by ~90% (Figure 2a). In the following step, a dose–response curve was determined for the inhibition of VEGFR-2 by Thio-Iva (0.003–30 µM) (Figure 2b). In the cell-free kinase assay, the IC_50_ value of Thio-Iva amounted to 3.31 µM. 

To confirm VEGFR-2 inhibiting effects on the cellular level, the dephosphorylating effects of Thio-Iva and Thio-Dam were determined in HuH-7 and SNU-449 cells (Figure 2c,d). Both compounds showed a dose-dependent suppression of VEGFR-2 phosphorylation in both cell lines, with Thio-Iva being more effective as compared to Thio-Dam. 

#### 3.1.3. Antiproliferative Activity of Thio-Iva and Thio-Dam in HCC Cells

**Figure 3:**
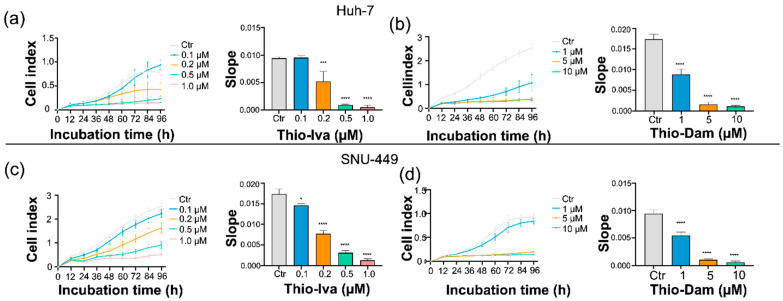
Real-time proliferation detection by iCELLigence. Dose-dependent effects of Thio-Iva and Thio-Dam on the cell index (CI) and its slope over time in Huh-7 (**a**,**b**) and SNU-449 cells (**c**,**d**). Statistical significance * *p* < 0.05, *** *p* < 0.001, and **** *p* < 0.0001 by ordinary one-way ANOVA as compared to untreated control. All results were expressed as means ± SEM of 3 independent experiments.

In order to further evaluate the dynamic effects of Thio-Iva and Thio-Dam on the proliferation of HCC, the iCELLigence system monitoring cell proliferation in real time was used. In the control group, both Huh-7 (Figure 3a) and SNU-449 (Figure 3c) cells showed vigorous growth, as shown by the cell index (CI), which continued to increase over time. By contrast, when treated with Thio-Iva or Thio-Dam (0.1–1.0 μM), both cell lines showed a dose-dependent reduction in the increase of the CI values, indicating a dose-dependent reduction of cell proliferation.

Calculating the slope of the cell index that reflected the proliferation dynamics over time revealed a dose-dependent and highly significant decline to almost zero which came after incubation with the rising concentrations of Thio-Iva and Thio-Dam, both in Huh-7 (Figure 3b) as well as in SNU-449 (Figure 3d) cells, thus impressively showing the antiproliferative effect of both compounds. Furthermore, the CI graphs also revealed that the onset of Thio-Iva-induced growth inhibition occurred after ~24 h, while that of Thio-Dam had already occurred after only ~12 h.

**Figure 4:**
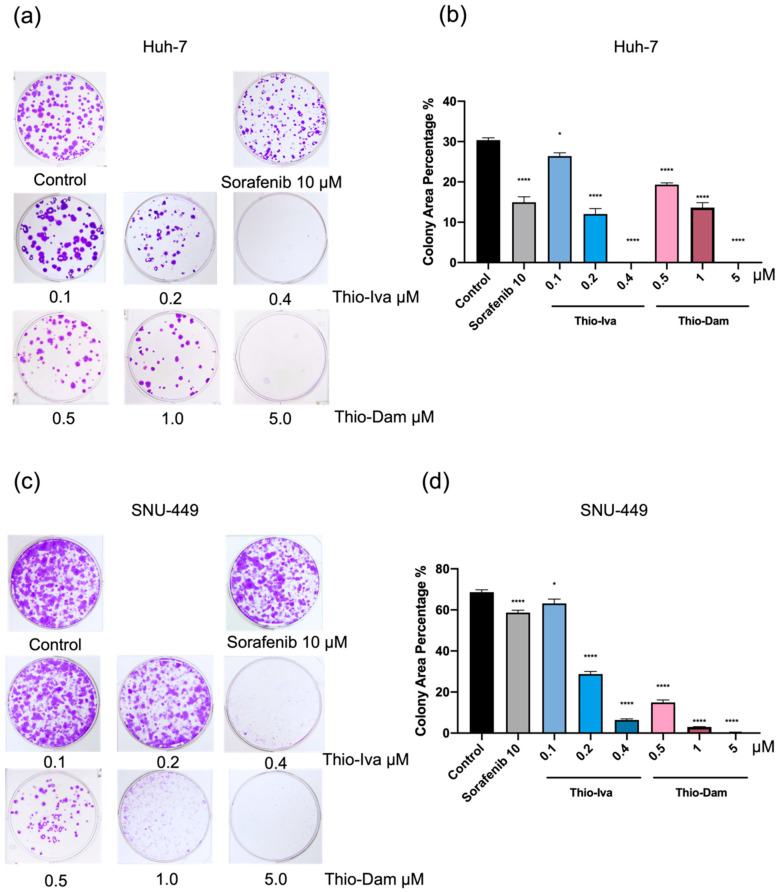
Clonogenic growth. Treatment of Huh-7 (**a**,**b**) and SNU-449 cells (**c**,**d**) with Thio-Iva, Thio-Dam, and sorafenib. The percentage of area occupied by the colonies was quantified using ImageJ software 14 days after plating. * *p* < 0.05 and **** *p* < 0.0001 by ordinary one-way ANOVA compared to control (untreated). All results were expressed as means ± SEM of ≥3 independent experiments.

In line with the iCELLigence proliferation data, long-term surveys (14 days) employing clonogenic assays also yielded a highly significant and dose-dependent reduction in the colony formation of Huh-7 (Figure 4a,b) and SNU-449 (Figure 4c,d) cells by >90% after Thio-Iva (0.1–0.4 µM) and Thio-Dam (0.5–5 µM) treatment, respectively. In both cell models, the anti-clonogenic effects of Thio-Iva and Thio-Dam exceeded by far the effect of the clinically relevant kinase inhibitor sorafenib (10 µM) (Figure 4b,d). 

#### 3.1.4. Unspecific Cytotoxicity

**Figure 5:**
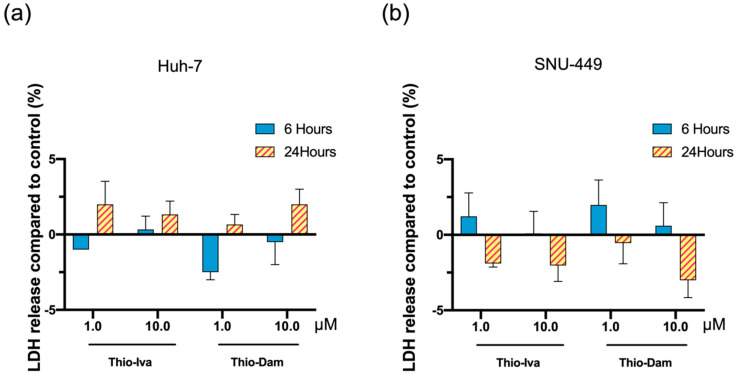
Cytotoxic effects of Thio-Iva and Thio-Dam. Release of lactate dehydrogenase (LDH) after incubation of Huh-7 (**a**) and SNU-449 (**b**) cells with 1 and 10 µM of Thio-Iva and Thio-Dam for 6 and 24 h, respectively. LDH release was not significantly altered when compared to untreated controls (set to 0%), indicating that unspecific toxicity did not contribute to the observed effects. Means ± SD of *n* = 3 independent experiments.

Unspecific cytotoxicity was evaluated by measuring LDH release into the supernatant of the Huh-7 (Figure 5a) and SNU-449 (Figure 5b) cell cultures after incubation for 6 and 24 h with Thio-Iva and Thio-Dam (1 and 10 µM), respectively. An increase of LDH levels in the supernatant indicates the nonspecific damage of cell membranes, which are not permeable to LDH in their undamaged state. However, even upon treatment with a high concentration of 10 µM, neither Thio-Iva nor Thio-Dam induced statistically significant increases in cytotoxicity after 6 h or 24 h, indicating that both compounds do not affect cell membrane integrity. Thus, an induction of immediate cytotoxicity is unlikely to account for the observed antiproliferative effects of the novel inhibitors.

#### 3.1.5. Apoptosis Induction and Regulation in HCC Cells

Apoptosis is the most prominent form of programmed cell death, which is mediated by the activation of effector caspases-3, -6, and -7. As procaspases, these proteases are usually inactive in non-malignant cells, which, however, can undergo autolytic activation upon stimulation to form active caspases. Among these caspases, caspase-3 is responsible for most proteolytic processes during apoptosis. Therefore, the detection of activated caspase-3 is a common marker for apoptosis [25]. 

**Figure 6:**
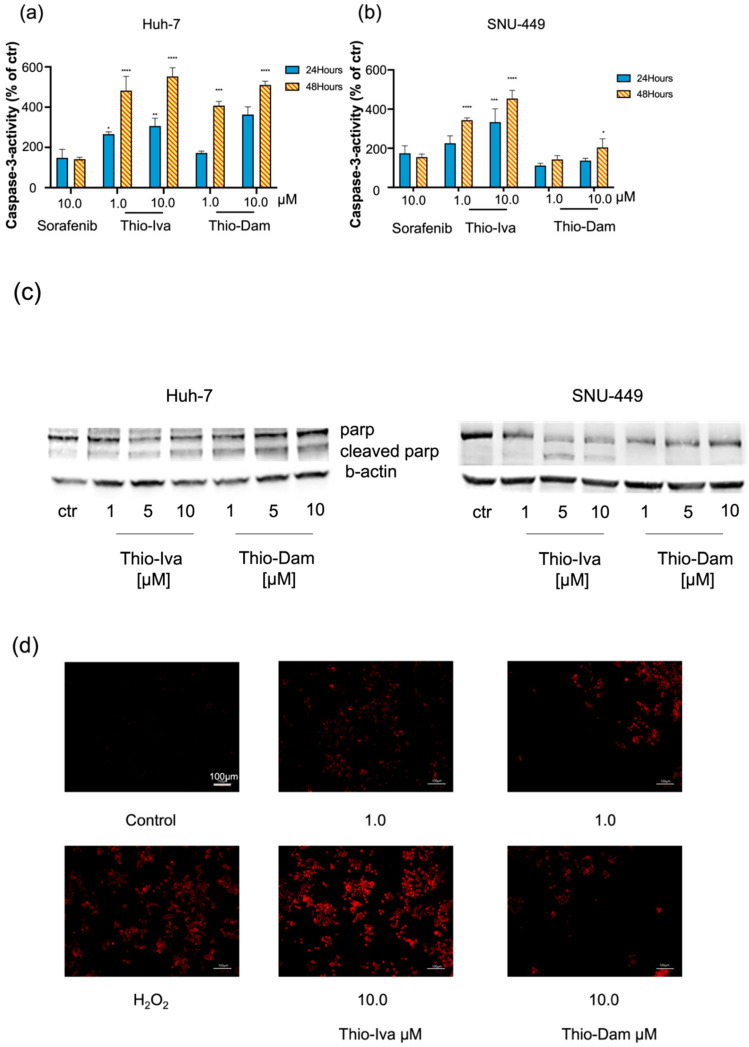
Induction of Apoptosis. (**a**) Dose- and time-dependent caspase-3 induction in Huh-7 cells after treatment with 1 and 10 µM of Thio-Iva and Thio-Dam and 10 µM sorafenib for 24 h and 48 h. (**b**) Dose- and time-dependent caspase-3 induction in SNU-449 cells after treatment with 1 and 10 µM of Thio-Iva and Thio-Dam and 10 µM sorafenib for 24 h and 48 h. Results are given as means ± SEM of *n* = 3 independent experiments. * *p* < 0.05, ** *p* < 0.01, *** *p* < 0.001, and **** *p* < 0.0001 by ordinary one-way ANOVA compared to untreated controls. (**c**) Representative Western blot results out of *n* = 3 independent experiments, showing PARP and cleaved PARP expression change by treatment, induced in Huh-7 and SNU-449 cells after 48 h. β-actin was used as loading control. (**d**) Detection of ROS induction by Thio-Iva and Thio-Dam in HCC cells after 24 h. H_2_O_2_ served as a positive control. Scale bar, 100 µm.

Treatment with Thio-Iva and Thio-Dam led to a significant and dose-dependent increase in caspase-3 activity in Huh-7 and SNU-449 cells, which was even stronger than those of sorafenib (10 µM). Thio-Iva and Thio-Dam showed significant dose- and time-dependent increases in caspase-3 activity. At 10 µM, Thio-Iva led to an approximately 3-fold increase when compared with untreated cells, and a 5.5-fold increase after 48 h. For SNU-449, a 3-fold increase was also seen after 24 h, and even a 5.5-fold increase after 48 h. Analogously, Thio-Dam (10 μM) showed a 3.5-fold increase in Huh-7 after 24 h, and a 5-fold increase after 48 h, but only a 2-fold increase was observed for SNU-449 after 48 h (Figure 6a,b). 

However, both of the thiophene derivatives induced a more pronounced caspase-3 activation in Huh-7 and SNU-449 cells than sorafenib (10 µM). Western blot analyses revealed that Thio-Iva induced apoptosis so as to promote poly-(ADP-ribose)-polymerase (PARP) cleavage in treated HCC cells (Figure 6c), while the effect of Thio-Dam was less pronounced in SNU-449 cells or was even absent in Huh-7 cells. 

Increased formation of reactive oxygen species (ROS) is a cell damage mechanism that plays an important role in cancer development and is also known as a trigger of mitochondria-driven apoptosis. The ROS-specific dye CellROX orange was applied to detect Thio-Iva and Thio-Dam-induced ROS formation in the cytoplasm of Huh-7 cells. After incubation for 24 h, a dose-dependent induction of ROS formation was observed in the cytoplasm of treated cells (Figure 6d).

#### 3.1.6. Cell-Cycle Regulation

**Figure 7:**
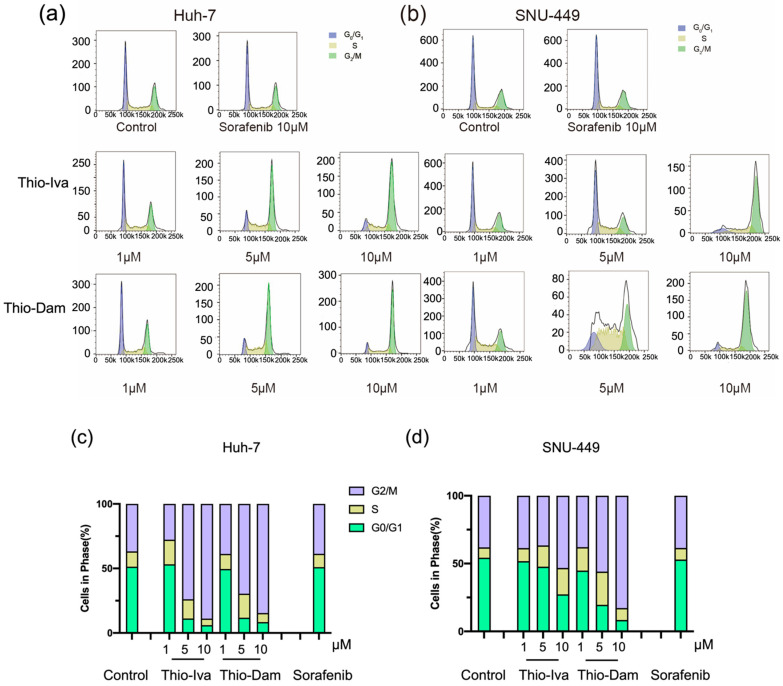

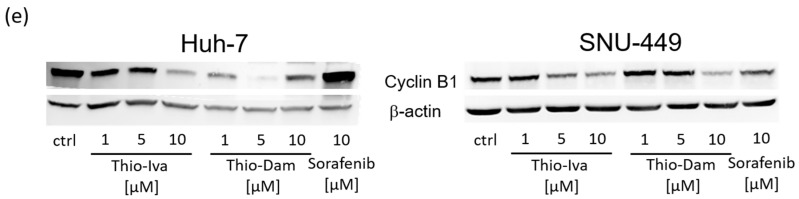
Flow cytometry revealed that Thio-Iva and Thio-Dam induced a pronounced G2/M arrest after 48 h in Huh-7 (**a**) and SNU-449 (**b**) cells. Quantification of the rate of the entire cell cycle; histogram shows average results for Huh-7 (**c**) and SNU-449 (**d**). All results are expressed as means ± SEM of *n* = 3 independent experiments. (**e**) Representative Western blots of *n* = 3 independent experiments showing cyclin B1 expression change in Huh-7 and SNU-449 cells upon treatment for 48 h. ß-actin was used as loading control.

The impact of Thio-Iva and Thio-Dam on the cell cycle of Huh-7 and SNU449 cells was determined by flow cytometry. Cells that were treated with 1, 5, and 10 µM of Thio-Iva and Thio-Dam for 48 h showed a dose-dependent and pronounced arrest in the G2/M phase of the cell cycle and a concomitant decrease of cells in the G0/1- and S-phases (Figure 7a–d). By contrast, sorafenib (10 µM) failed to induce a pronounced cell cycle arrest in Huh-7 or SNU-449 cells. 

Cyclin B1, a key component in the control of cell cycle progression from the G2 to the M phase, has been implicated in the tumorigenesis and the development of malignancy. Cells suppress and degrade the cell cycle promoter, cyclin B1, in order to escape mitosis [28]. The expression of cyclin B1 was determined to further decipher the molecular mechanism of the G2/M phase blockade by Thio-Iva and Thio-Dam. After 48 h of incubation, both Thio-Iva and Thio-Dam down-regulated cyclin B1 in a dose-dependent manner (Figure 7e), thereby fitting to the flow cytometry findings on a G2/M arrest by the thiophene derivatives in the Huh-7 and SNU-449 cells. The lack of sorafenib to suppress cyclin B1 expression (Figure 7e) corroborates the observation that sorafenib did not induce an appreciable G2/M arrest in either HCC cell line (Figure 7c,d).

#### 3.1.7. Inhibition of Cell Migration

**Figure 8:**
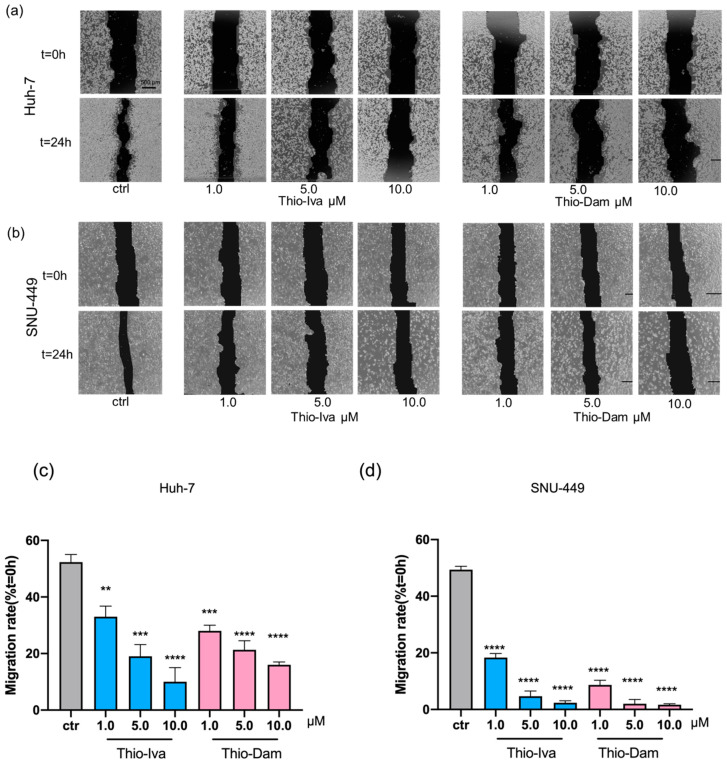
Antimigratory effects of Thio-Iva and Thio-Dam in Huh-7 and SNU-449. (**a**) Representative images of antimigratory effects of Thio-Iva and Thio-Dam (1–10 μM) in Huh-7 cells after 24 h. (**b**) Representative images of antimigratory effects of Thio-Iva and Thio-Dam in SNU-449 cells after 24 h. (**c**,**d**) Quantification of the migration rate (in %) of SNU-449 cells after incubation with Thio-Iva and Thio-Dam. ** *p* < 0.01, *** *p* < 0.001, and **** *p* < 0.0001 by ordinary one-way ANOVA compared to untreated controls. Results are given as means ± SEM of = 3 independent experiments. Scale bar, 500 µm.

It is mandatory to block the migration and spreading of tumor cells in order to prevent the formation of metastases. Thus, new compounds which inhibit tumor cell migration are of particular interest for the development of new anticancer agents. Wound healing (scratch) assays were performed to investigate the motility of HCC cells treated with Thio-Iva and Thio-Dam (Figure 8). In order to ensure that the scratched gap is filled by migration and not by proliferation, cells were cultured in FBS-free medium for 24 h. The migration rate of untreated Huh-7 control cells was ca. 52.3% after 24 h, while Huh-7 cells treated with Thio-Iva (1, 5, and 10 μM) acted in a dose-dependent way and showed reduced Huh-7 cell migration rates of 33.0%, 19.0%, and 10.0%, respectively. Thio-Dam (1, 5, and 10 μM) showed migration rates of 28.0%, 21.3%, and 16.0%, respectively, which are similar to the rates of Thio-Iva (Figure 8a,c). In SNU-449 cells, the migration rate of untreated control cells was 49.3%, and upon Thio-Iva treatment (1, 5, 10 µM) dropped dose-dependently to 18.3%, 4.6%, and 2.3%, respectively. Comparable results were found for Thio-Dam treatment (1, 5, 10 µM), which resulted in a drop in SNU-449 migration rates of 8.7%, 2.0%, and 1.7%, respectively (Figure 8b,d).

#### 3.1.8. Antiangiogenic Effects of Thio-Iva and Thio-Dam In Vitro and In Vivo

**Figure 9:**
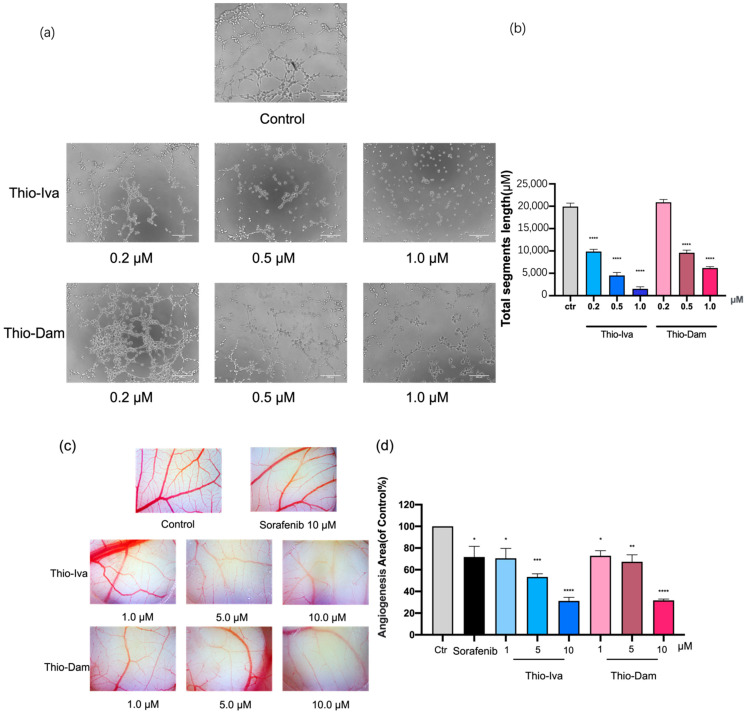
In vitro and in vivo effects of Thio-Iva and Thio-Dam on angiogenesis. (**a**) Representative images of tube formation assay with EA.hy926 cells. Thio-Iva and Thio-Dam (0.2–1 µM) were applied for 6 h. (**b**) Tube formation of EA.hy926 cells was quantified and depicted as changes in total segment length using ImageJ software. (**c**) CAM assay showing inhibition of angiogenesis in vivo: Representative examples of CAM were taken from a typical experiment. Untreated control is the area outside the silica ring. Inside the silicone ring, the surface was treated with different concentrations of Thio-Iva and Thio-Dam for 48 h. (**d**) Image-Pro Plus software was used in analysis for blood vessel area quantification (compared to control in %). * *p* < 0.05, ** *p* < 0.01, *** *p* < 0.001, and **** *p* < 0.0001 by ordinary one-way ANOVA compared to control (untreated area). Results are given as means ± SEM of = 3 independent experiments.

The effects of Thio-Iva and Thio-Dam on angiogenesis were investigated both in vitro and in vivo. Initially, in vitro tube formation assays with endothelial EA.hy926 cells were performed (Figure 9a). Treatment with Thio-Iva led to a strong inhibition of tube formation, with 51% at 0.2 µM and even up to 92% at 1 μM. The effect of Thio-Dam was less pronounced. However, at a dose of 1 μM, Thio-Dam induced an almost 70% inhibition of tube formation (Figure 9b).

In addition, chicken CAM assays were employed to determine the in vivo anti-angiogenic effects of Thio-Iva and Thio-Dam. Both compounds showed considerable reductions of angiogenesis in a dose-dependent way, which were stronger than those of sorafenib, an established anti-angiogenic drug for the treatment of HCC. The vessels displayed morphological irregularities in response to the treatment with Thio-Iva and Thio-Dam, which were not observed in the untreated controls (Figure 9c,d).

#### 3.1.9. Antineoplastic Effects of Thio-Iva and Thio-Dam In Vivo 

**Figure 10:**
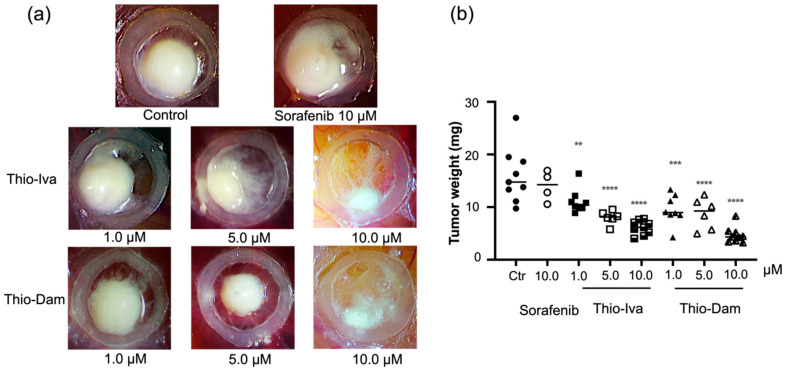
In vivo antineoplastic effects of Thio-Iva and Thio-Dam on Huh-7-derived HCC micro tumors. (**a**) Representative images of Thio-Iva and Thio-Dam-treated microtumors grown on the CAM of fertilized chicken eggs after 72 h. Sorafenib was additionally applied as a clinically relevant HCC therapeutic. PBS-treated microtumors served as controls. (**b**) Tumor weight analysis. ** *p* < 0.01, *** *p* < 0.001, and **** *p* < 0.0001 by ordinary one-way ANOVA compared to controls. Results are shown as means ± SEM of = 3 independent experiments.

CAM assays were performed to demonstrate the effects of Thio-Iva and Thio-Dam on HCC tumor growth in vivo. Micro tumors of Huh-7 cells were grown on the CAM and were treated with Thio-Iva (1–10 μM) and Thio-Dam (1–10 μM) for 72 h. A dose-dependent and highly significant reduction of tumor growth was observed, and the tumor weight decreased by up to 62% (Thio-Iva) and 71% (Thio-Dam), respectively, as compared to PBS-treated controls (Figure 10a,b). Interestingly, sorafenib did not induce a significant reduction of Huh7 microtumor growth in these experiments. It is noteworthy that no increased embryonic lethality rate or signs of developmental retardation was observed in the treated eggs, indicating the good tolerability of the novel compounds—a finding that corroborates the absence of unspecific cytotoxic effects of Thio-Iva and Thio-Dam in Huh-7 and SNU-449 cells in the respective LDH-release assays (Figure 5).

## 4. Discussion

Hepatocellular carcinoma is the fourth leading cause of cancer death in the world, which accounts for more than 80% of global primary liver cancers [29]. In recent years, significant progress has been made in terms of surgical treatment, interventional therapy, and radiotherapy for patients suffering from early HCC [3,30]. However, medical treatment methods for patients with advanced HCC showed only marginal improvements in efficacy [31]. Therefore, there is an urgent need for new drugs for the treatment of advanced HCC. Recently, we introduced a series of 2-(thien-2-yl)-acrylonitriles with different aryl substituents (such as hydroxyl and alkoxy, dialkylamine, and halogen) as protein kinase inhibitory compounds with antineoplastic potency [21]. 

In the present study, the anti-tumor effects of Thio-Iva and Thio-Dam, the two most effective compounds of the series, were further elaborated in terms of their anticancer properties in two HCC cell models. Their underlying modes of action were deciphered as well as their antineoplastic and anti-angiogenic effects in vivo.

Aggressive tumors are characterized by a sustained proliferation of tumor cells. We showed that the compounds Thio-Iva and Thio-Dam exert strong antiproliferative effects on HCC cells in a dose- and time-dependent manner, which even exceeded those of the clinically approved multi-kinase inhibitor sorafenib. In addition, real-time proliferation monitoring revealed that Thio-Iva and Thio-Dam exerted their growth inhibitory effects after only 12 and 24 h, respectively. In addition, colony formation assays showed the long-term anti-proliferative effects of the novel thiophene derivatives, while the anticancer effects of Thio-Iva and Thio-Dam were not based on the induction of unspecific cytotoxicity but involved the induction of apoptosis and cell cycle arrest as specific modes of action. 

As the evasion of apoptosis by cancer cells is one of the leading causes of uncontrolled tumor cell growth, the acquisition of anti-apoptotic features during carcinogenesis is regarded as one of the hallmarks of cancer [32]. The cysteine protease caspase-3 is the most important executioner caspase of cellular apoptosis [33], and thus its induction by Thio-Iva and Thio-Dam was determined as an unequivocal sign of apoptosis induction in Huh-7 and SNU-449 cells. Treatment with Thio-Iva and Thio-Dam in a time- and dose-dependent manner induced caspase-3 activity, which even exceeded by far those of sorafenib. The increase of ROS levels can promote the dissipation of mitochondrial membrane potential, can cause organ dysfunction, and can trigger mitochondria-driven apoptosis. Moreover, excessive ROS levels are also related to DNA damage [34]. Thio-Iva and Thio-Dam were shown to induce pronounced increases in cytoplasmic ROS levels. Mitochondria are the main source of ROS in cells and the most severely affected organelles of cellular stress [35]. In order to link the Thio-Iva and Thio-Dam-induced rise in cytoplasmic ROS to the mitochondria-driven apoptosis of HCC cells, we demonstrated in this study that the acute burst of ROS in mitochondria specifically causes cell apoptosis and subsequently activates caspase-3. Western blotting showed that PARP, the substrate of caspase-3, was reduced and cleaved into an N-terminal 89 kDa fragment in the Huh-7 cell line incubated with Thio-Iva. Albeit less pronounced, Thio-Dam treatment also decreased PARP expression, at least in SNU-449 cells. 

In terms of DNA damage, blocking cell-cycle checkpoints can lead to genome instability and subsequent cell death. G2 abolition prevents cancer cells from repairing DNA damage, forcing them to enter the M phase and the so-called “mitotic catastrophe” as well as apoptosis [36,37]. The G2 checkpoint has become an attractive therapeutic target for anticancer therapy. Flow cytometry analyses revealed a dose-dependent G2/M phase arrest in Huh-7 and SNU-449 cells treated with Thio-Iva and Thio-Dam. These compounds meet the criteria of ideal G2 checkpoint inhibitors, which selectively target molecules that do not participate in the G1 checkpoint or S phase checkpoints [38]. The precise regulation of cyclin B1 is essential for the onset of mitosis and for checkpoint control. It was also shown to regulate cell cycle transition from the G2 to the M phase [39,40]. Thio-Iva and Thio-Dam significantly suppressed cyclin B1 in both cell lines after 48 h, while treatment with sorafenib only had slight effects on cyclin B1 expression, which is in line with our findings from the flow cytometry experiments. More and more evidence suggest that cyclin B1 is highly expressed in several tumors, and its effects were correlated with tumor proliferation, invasion, and apoptosis [40,41]. The distinct suppression of cyclin B1 by Thio-Iva and Thio-Dam can explain the pronounced pro-apoptotic activities of these compounds.

As a malignant, hyper-vascularized solid tumor, HCC can be treated by inhibiting angiogenesis. Tumor angiogenesis is regarded as another hallmark of cancer [32] and may thus be an important target in HCC treatment. Sorafenib inhibits multiple receptor tyrosine kinases such as the VEGFR and PDGFR signaling pathways and has been the first-line drug in the treatment of advanced HCC for a long time [2]. However, a considerable number of HCC patients had to stop sorafenib treatment due to unbearable side effects or drug resistance. Therefore, some other multi-kinase inhibitors such as brivanib, sunitinib, and linifanib were studied, but these failed in phase III trials [11,42,43]. VEGFR-2 is a transmembrane receptor tyrosine kinase that functions in both physiological and pathological angiogenesis. The activation of VEGFR-2 promotes endothelial cell invasion, migration, proliferation, and angiogenesis [44,45]. However, the VEGFR-2 inhibitor ramucirumab also failed to reach the end point in a recent phase III trial [10]. Thio-Iva and Thio-Dam were recently described as novel multi-kinase inhibitors with preferential VEGF Receptor inhibition [21]. Endothelial cells are involved in angiogenesis and proliferate to provide the cells required to form new blood vessels. After proliferation, endothelial cells reorganize into a three-dimensional tubular structure [46]. Our in vitro experiments demonstrated that Thio-Iva and Thio-Dam affect EA.hy926 cell tube formation even at low concentrations. In particular, cells treated with Thio-Iva displayed no tubular structure at all. In addition, CAM assays showed that Thio-Iva and Thio-Dam also reduced angiogenesis in vivo and to a higher degree than sorafenib. Vessels in treated CAMs displayed visible morphological irregularities (Figure 7b).

As a prerequisite for invasion and metastasis, the migration of tumor cells is regarded as another hallmark of cancer [32]. HCC cells treated with Thio-Iva and Thio-Dam showed significantly decreased cell migration, indicating that both compounds exert anti-metastatic properties. 

The CAM assay is an established model for testing anti-tumor compounds in vivo and can be used as a template for growing micro tumors from human cancer cell lines [47]. We showed that Thio-Iva and Thio-Dam reduce the growth of HCC tumors grown on CAMs, thus confirming their considerable antineoplastic effects in vivo. Both compounds were well-tolerated and did not exhibit embryo toxicity or developmental delay. The effects of the novel thiophene-based compounds were also notably more effective than the treatment with the clinically established multi-kinase inhibitor sorafenib. 

## 5. Conclusions

In conclusion, we demonstrated the pronounced antiproliferative, apoptosis-inducing, antimigratory, and cell cycle-arresting properties of the two novel 2-(thien-2-yl)-acrylonitrile kinase inhibitors in HCC cells. In addition, Thio-Iva and Thio-Dam showed significant antitumor and antiangiogenic effects as well as excellent in vivo tolerance. The novel compounds were shown to effectively attack HCC cells in cellular processes and features that are acquired during carcinogenesis and which are referred to as hallmarks of cancer. Our results show that Thio-Iva and Thio-Dam may provide new and valuable options for the treatment of hepatocellular carcinoma in the future. Hence, future investigations of these promising kinase inhibitors are warranted.

## Figures and Tables

**Table 1:** Determination of IC_50_ values (µM) of test compounds for the Huh-7 and SNU-449 HCC cell lines after incubation for 48 h.

Compounds	Huh-7	SNU-449
Thio-Iva	0.29 ± 0.18	0.53 ± 0.32
Thio-Dam	0.81 ± 0.26	1.64 ± 0.51
Thio-Anis	1.20 ± 0.42	>8
Thio-AniMe	1.85 ± 0.21	>8
Sorafenib	2.50 ± 0.14	>8

## Data Availability

Not applicable.
